# Stimulation of the calcium‐sensing receptor induces relaxations through CGRP and NK1 receptor‐mediated pathways in male rat mesenteric arteries

**DOI:** 10.14814/phy2.16125

**Published:** 2024-06-20

**Authors:** Simonette R. E. Carlton‐Carew, Harry Z. E. Greenberg, Iain A. Greenwood, Anthony P. Albert

**Affiliations:** ^1^ Vascular Biology Section, Cardiovascular & Genomics Research Institute St. George's, University of London London UK

**Keywords:** calcitonin gene‐related peptide, calcium‐sensing receptor, perivascular sensory nerves, substance P, vascular smooth muscle

## Abstract

Stimulation of the calcium‐sensing receptor (CaSR) regulates vascular contractility, but cellular mechanisms involved remain unclear. This study investigated the role of perivascular sensory nerves in CaSR‐induced relaxations of male rat mesenteric arteries. In fluorescence studies, colocalisation between synaptophysin, a synaptic vesicle marker, and the CaSR was present in the adventitial layer of arterial segments. Using wire myography, increasing external Ca^2+^ concentration ([Ca^2+^]_o_) from 1 to 10 mM induced vasorelaxations, previously shown to involve the CaSR, which were inhibited by pretreatment with capsaicin. [Ca^2+^]_o_‐induced vasorelaxations were partially reduced by the calcitonin gene‐related peptide (CGRP) receptor blockers, CGRP 8–37 and BIBN 4096, and the neurokinin 1 (NK1) receptor blocker L733,060. The inhibitory effect of CGRP 8–37 required a functional endothelium whereas the inhibitory action of L733,060 did not. Complete inhibition of [Ca^2+^]_o_‐induced vasorelaxations occurred when CGRP 8–37 and L733,060 were applied together. [Ca^2+^]_o_‐induced vasorelaxations in the presence of capsaicin were abolished by the ATP‐dependent K^+^ channel (K_ATP_) blocker PNU 37883, but unaffected by the endothelium nitric oxide synthase (eNOS) inhibitor L‐NAME. We suggest that the CaSR on perivascular sensory nerves mediate relaxations in rat mesenteric arteries via endothelium‐dependent and ‐independent mechanisms involving CGRP and NK1 receptor‐activated NO production and K_ATP_ channels, respectively.

## 1 INTRODUCTION

It is well‐established that activation of the calcium‐sensing receptor (CaSR), a member of the Class C family of G‐protein‐coupled receptors (GPCRs), in the parathyroid gland by an increase in plasma Ca^2+^ concentration regulates parathyroid hormone synthesis and secretion to maintain plasma Ca^2+^ homeostasis through intestinal Ca^2+^ absorption, renal Ca^2+^ excretion and bone remodeling (Brown & MacLeod, 2001; Hannan et al., 2018). It is also increasingly apparent that the CaSR is present in tissues previously defined as non‐calciotropic such as the vasculature (Guo et al., 2018; Smajilovic et al., 2011; Weston et al., 2011), although recent evidence indicates that the CaSR in the vasculature may indeed modulate Ca^2+^ homeostasis (Hannan et al., 2018; Schepelmann et al., 2022). Stimulation of the CaSR in the vasculature is considered possible as localized extracellular Ca^2+^ concentration ([Ca^2+^]_o_) is likely to vary sufficiently compared to maintained plasma Ca^2+^ homeostasis due to active Ca^2+^ extrusion mechanisms such as the plasmalemmal Ca^2+^‐ATPase and Na^+^‐Ca^2+^ exchanger present on vascular smooth muscle cells (VSMCs) and vascular endothelial cells (VECs) (Dora et al., 2008).

In the vasculature, the CaSR is proposed to be expressed in perivascular sensory nerves, VSMCs, and VECs and there is significant evidence that stimulation of the CaSR induces vasorelaxations (Awumey et al., 2008, 2013; Bukoski et al., 1997; Carlton‐Carew et al., 2024; Greenberg et al., 2017, 2019; Greenberg, Jahan, et al., 2016; Greenberg, Shi, et al., 2016; Ishioka & Bukoski, 1999; Loot et al., 2013; Mupanomunda et al., 1998, 1999; Tang et al., 2016; Thakore & Ho, 2011; Wang & Bukoski, 1998, 1999; Weston et al., 2005, 2008), vasoconstrictions (Li et al., 2011; Schepelmann et al., 2016; Wonneberger et al., 2000), and biphasic responses (Ohanian et al., 2005). Possible involvement of the CaSR in excessive vasocontraction or vasodilatation may indicates it is a therapeutic target for cardiovascular conditions such as systemic and pulmonary hypertension and septic shock (Guo et al., 2018; Sood et al., 2022; Zhang et al., 2024).

CaSR‐induced regulation of vascular contractility has been linked to the expression of CaSR on different cell types and involving different cellular mechanisms, with the potential for species and vascular bed differences. Stimulation of the CaSR on perivascular sensory nerves is reported to induce relaxation of rat mesenteric arteries via endothelium‐dependent and ‐independent pathways involving synthesis and release of cytochrome P_450_ and phospholipase A_2_ metabolites and anandamide, which activate large conductance Ca^2+^‐activated K^+^ channels (BK_Ca_) in VSMCs to produce membrane hyperpolarization (Awumey et al., 2008; Bukoski et al., 1997, 2002; Ishioka & Bukoski, 1999; Mupanomunda et al., 1998, 1999; Wang & Bukoski, 1998, 1999). Stimulation of the CaSR on VECs have been proposed to induce relaxations in rat mesenteric arteries through activation of intermediate Ca^2+^‐activated K^+^ channels (IK_Ca_) and hyperpolarization of VSMCs (Thakore & Ho, 2011; Weston et al., 2005, 2008). In mouse aorta, CaSR‐mediated relaxations involve stimulation of endothelium nitric oxide synthase (eNOS) and nitric oxide (NO) production (Loot et al., 2013). Our studies in mouse and rabbit mesenteric arteries identified that activation of the CaSR on VECs induce relaxations via two separate endothelium‐dependent pathways involving stimulation of heteromeric TRPV4‐TRPC1 channels, eNOS stimulation, and NO generation which activates BK_Ca_ channels in VSMCs and stimulation of IK_Ca_ channels and endothelium‐derived hyperpolarization of VSMCs (Greenberg et al., 2017, 2019; Greenberg, Shi, et al., 2016). CaSR‐induced activation of Gq‐mediated pathways in VSMCs are linked to vasoconstriction (Li et al., 2011; Schepelmann et al., 2016; Wonneberger et al., 2000). Interestingly, inhibition of CaSR‐mediated vasorelaxations can reveal CaSR‐mediated vasoconstrictor responses indicating that CaSR‐induced effects on vascular contractility may involve a balance between relaxant and contractile mechanisms (Carlton‐Carew et al., 2024; Greenberg, Jahan, et al., 2016; Greenberg, Shi, et al., 2016).

In a recent study, we re‐evaluated the effect of stimulating the CaSR in rat mesenteric arteries and showed that stimulation of the CaSR by increasing [Ca^2+^]_o_ induced relaxations through two pathways: an endothelium‐dependent pathway involving NO synthesis and activation of BK_Ca_ channels in VSMCs and an endothelium‐independent pathway involving ATP‐dependent K^+^ channels (K_ATP_) in VSMCs. In the present study, we extended this work to investigate the role of the CaSR expressed in perivascular sensory nerves in producing [Ca^2+^]_o_‐induced vasorelaxations. We propose for the first time that stimulation of the CaSR on capsaicin‐sensitive perivascular sensory nerves has an important role in mediating [Ca^2+^]_o_‐induced vasorelaxations through calcitonin gene‐related peptide (CGRP) receptor (known to be composed of calcitonin receptor‐like receptor (CLR) and receptor activity modifying proteins 1 (RAMP1), Liang et al., 2018) and possibly NK1 receptor‐mediated pathways, with CGRP receptors likely coupled to endothelium‐dependent NO production and NK1 receptors to stimulation of K_ATP_ channels in VSMCs via an endothelium‐independent pathway.

## 2 METHODS AND MATERIALS

### 2.1 Ethical approval

All animal procedures were carried out in accordance with guidelines laid down by St. George's, University of London Animal Welfare Committee, and conform with the principles and regulations described by the Service Project Licence: 70/8512 and to the principles and regulations described by Grundy (2015). Male Wistar rats (aged 10–12 weeks and weighing between 210 and 280 g) were used for the purpose of the study. Rats were supplied from Charles River Ltd (Margate, Kent, UK) and housed and maintained in standard‐sized plastic cages at the Biological Research Facility at St. George's, University of London under a 12:12 h light/dark photocycle at 18–22°C and 50 ± 10% relative humidity, with freshwater and laboratory rodent maintenance diet (Special Dietary Services, UK) ad libitum.

Rats were culled by cervical dislocation and death was confirmed by severance of the femoral artery in accordance with Schedule I of the UK Animals (Scientific Procedures) Act of 1986. First‐order branches of superior mesenteric arteries were dissected and cleaned of adherent tissue in ice‐cold Krebs–Henseleit physiological salt solution (PSS) containing (mM): NaCl 118, KCl 4.7, MgSO_4_ 1.2, KH_2_PO_4_ 1.2, NaHCO_3_ 25, CaCl_2_ 1, D‐glucose 10.

### 2.2 Immunofluorescence in arterial segments

Dissected first‐order arterial segments were fixed with 4% (w/v) paraformaldehyde (PFA) in phosphate buffered saline solution (PBS) for 1 h at room temperature, and then washed three times in PBS for 10 min each. Next, the blocking buffer 20% (v/v) donkey serum in PBS with 0.2% (v/v) Triton was pipetted into the 24‐well plate containing the arterial segments and incubated with gentle agitation for 1 h. Mouse anti‐CaSR antibody (ab19347, 1:100, Abcam) and a rabbit anti‐synaptophysin antibody (ab32127, 1:100, Abcam) were dissolved in PBS containing 2% (v/v) donkey or goat serum and 0.2% (v/v) Triton, and pipetted (100 μL each) into the plates containing the arterial segments and placed at 4°C overnight. The following day, the microscope slides were washed three times at 10 min each with PBS, and then secondary antibodies Alexa Fluor 555‐conjugated donkey (A‐31570, 1:500) or goat (A‐21422, 1:500) anti‐mouse antibody and Alexa Fluor 546‐conjugated donkey anti‐rabbit antibody (A‐10040, 1:500) (ThermoFisher Scientific, Walham, MA, USA) dissolved in 2% (v/v) donkey or goat serum in PBS containing 0.2% (v/v) Triton was pipetted into the plates. A drop of DAPI (4′,6‐diamidino‐2‐phenylindole, cat 62248, ThermoFisher Scientific) was added to the plates and placed in a humidity chamber covered with an opaque cover and left in a dark room for 1 h 30 min at room temperature. Fluorescent and brightfield images of arterial segments were captured using the Zeiss LSM510 META Inverted confocal scanning laser microscope (Carl Zeiss, Jena, Germany). Excitation was produced by 546 or 555 nm lasers and delivered to the specimen via a Zeiss Apochromat ×10/×20 oil‐immersion objective. Emitted fluorescence was captured using LSM 510 software (release 3.2; Carl Zeiss), and images were produced using PowerPoint (Microsoft Office, Richmond, WA, USA).

### 2.3 Isometric tension recordings

Contractility of first‐order rat mesenteric arteries were measured using wire myography as previously described (Carlton‐Carew et al., 2024). Vessel segments of 2 mm in length were mounted in a wire myograph (Model 610 M; Danish Myo Technology, Aarhus, Denmark) and equilibrated at 37°C for 30 min in 5 mL of gassed (95% O_2_/5% CO_2_) PSS, pH 7.2. Vessels were then normalized to 90% of the internal circumference predicted to occur under a transmural pressure of 100 mmHg (Mulvany & Halpern, 1977). After normalization, vessels were left for 10 min and were then challenged with 60 mM KCl for 5 min. Endothelium integrity was assessed by stably precontracting vessels with 300 nM U46619 (cat 1932, R&D systems), a thromboxane A2 mimetic (concentration producing approximately 90% of maximal contraction), followed by the addition of 10 μM carbachol (CCh, C4382, Sigma‐Aldrich). Vessels in which CCh‐induced relaxations were ≥90% of precontracted tone were designated as having a functional endothelium. Endothelium was removed by rubbing the intima layer with a human hair and CCh‐induced relaxations of ≤10% of precontracted tone indicated successful removal. All vessel segments used contained a functional endothelium unless otherwise stated. Vessel segments were incubated for 30 min in fresh PSS solution containing 1 mM CaCl_2_ and then precontracted with the thromboxane receptor agonist 300 nM U46619. This was followed by cumulative additions of CaCl_2_, increasing [Ca^2+^]_o_ from 1 to 10 mM. Capsaicin (M2028, Sigma‐Aldrich) was added to the vessel segments 1 h before construction concentration‐response curves to [Ca^2+^]_o_. All other inhibitors were added to the vessel segments 30 min before generation of concentration‐response curves to [Ca^2+^]_o_. All relaxant responses are expressed as percentage relaxation of tension induced by 300 nM U64419. Data points on all graphs are mean values and error bars representing standard deviation. For each experiment *n* = number of animals used and numbers of vessel segments from these animals are shown in brackets. Individual cumulative concentration‐effect curves of [Ca^2+^]_o_ on U46619‐induced contractility were analyzed using a sigmoidal four‐parameter logistic equation using Graphpad Prism 6 software (GraphPad Software, Inc, San Diego, CA, USA) to extrapolate the effective concentration that produces 50% relaxation (EC_50_) or maximal effect at 10 mM [Ca^2+^]_o_ (*E*_max_). Mean EC_50_ and *E*_max_ values were compared using un‐paired Students *t*‐test. Mean cumulative responses to increasing [Ca^2+^]_o_ were analyzed by two‐way ANOVA followed by Bonferroni post hoc tests. *p* values are shown to 4 decimal points, with *p* < 0.05 taken as statistically significant. Statistical analysis and graphs were made using Graphpad Prism 6 and Origin, version 6.1 (MicroCal Software, Northampton, MA, USA).

### 2.4 Materials

We used a mouse anti‐CaSR antibody (Abcam, ab19347) and a rabbit anti‐synaptophysin antibody (Abcam, ab32127). The monoclonal mouse anti‐CaSR antibody used has been previously validated for the CaSR using siRNA (Mary et al., 2015), shRNA (Wei et al., 2015), and activating and knockdown CRISR technologies (Wu et al., 2019). All drugs were purchased from Sigma‐Aldrich (Sigma Chemical Co., Poole, UK), R&D Systems (Abingdon, UK) or Tocris (Tocris Biosciences, Bristol, UK), and dissolved in distilled water (d.H_2_O), dimethyl sulfoxide (DMSO), or ethanol (EtOH).

## 3 RESULTS

### 3.1 CaSRs are expressed in perivascular nerves in rat mesenteric arteries

**FIGURE 1:**
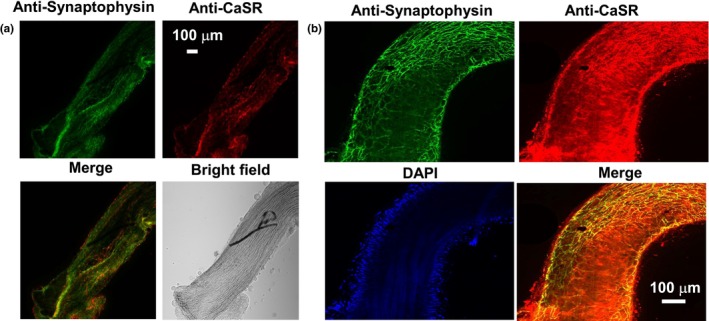
Expression of the CaSR in perivascular nerves of rat mesenteric arteries. (a) Representative immunofluorescence image (×10) from a first‐order rat mesenteric arterial segment showing co‐localisation staining with an anti‐synaptophysin antibody, as marker for synaptic terminals, and an anti‐CaSR antibody. (b) Immunofluorescence image from a different first‐order arterial segment under higher magnification (×20) showing that co‐localisation staining with anti‐synaptophysin and anti‐CaSR antibodies in mesh‐like structures at the adventitial layer.

In our initial experiments, we studied CaSR expression in perivascular nerves in segments of first‐order male rat mesenteric arteries using immunofluorescence techniques. Figure 1a,b shows that staining with an antibody for the synaptic terminal marker synaptophysin produced a mesh‐like network of perivascular nerves around the adventitial layer of arterial segments, which contained co‐localisation with the CaSR. In addition, Figure 1a,b also shows areas of arterial segments only stained for the CaSR which likely represent expression on VSMCs and VECs as previously described (Carlton‐Carew et al., 2024).

### 3.2 Pretreatment with capsaicin reduced [Ca^2+^]_o_‐induced relaxations of rat mesenteric arteries

To investigate the role of the CaSR on perivascular sensory nerves in regulating contractility of rat mesenteric arteries, we used wire myography and studied the effect of pretreatment with capsaicin on [Ca^2+^]_o_‐induced relaxation of precontracted tone evoked by the thromboxane receptor agonist U46619. Capsaicin pretreatment is an established method to investigate the role of perivascular sensory nerves in regulating vascular function, as it stimulates and desensitizes TRPV1 channels present on perivascular sensory nerve terminals and thus reduces the release of vasoactive substances such as CGRP, Sub P, and anandamide (Aalkjaer et al., 2021; Wescott & Segal, 2013).

**FIGURE 2:**
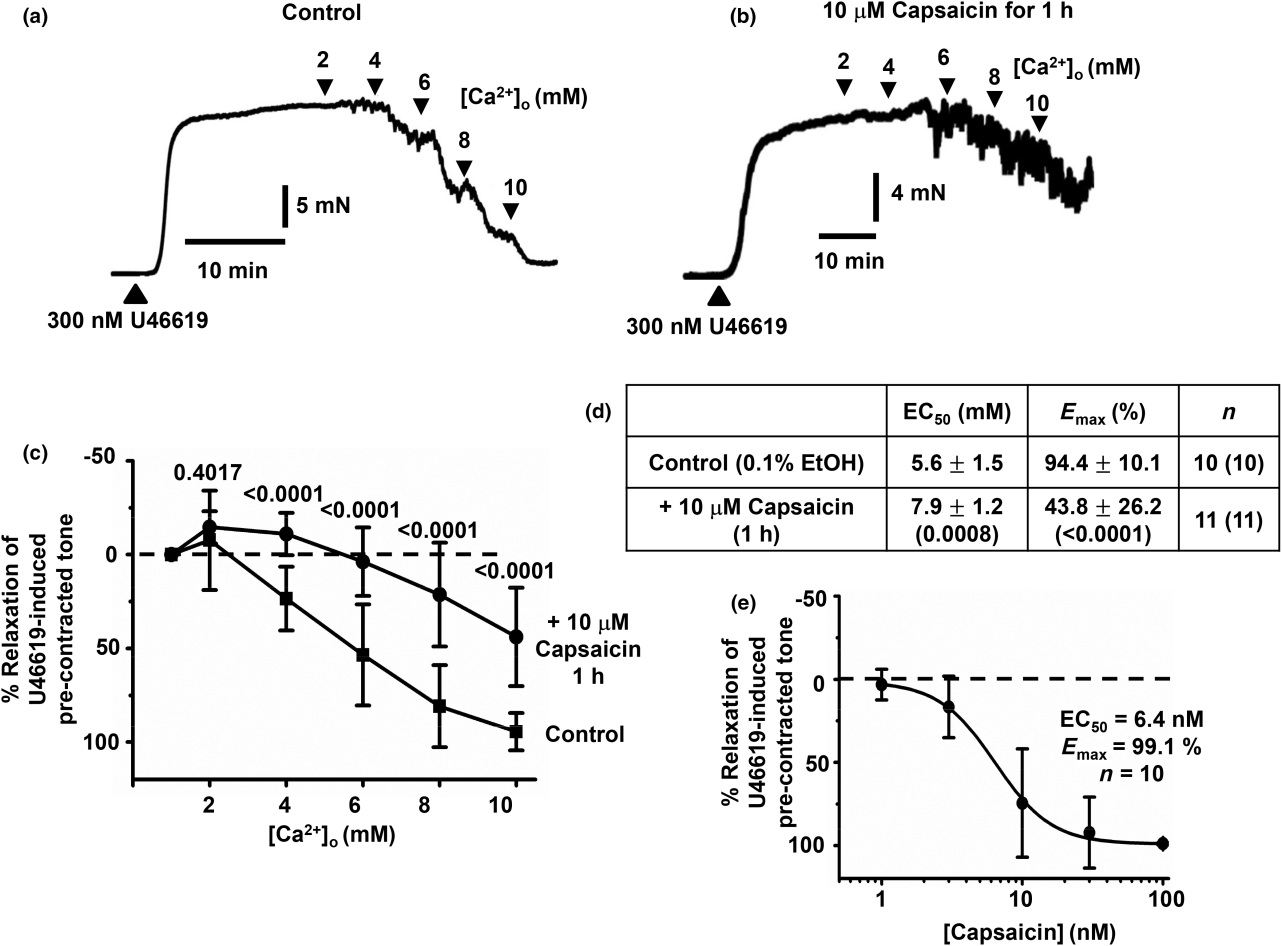
Effect of capsaicin on [Ca^2+^]_o_‐induced relaxations of precontracted tone in rat mesenteric arteries. (a and b) Representative records showing that increasing [Ca^2+^]_o_ induced relaxations of precontracted tone by 300 nM U46619 in first‐order arterial segments, which were reduced by pretreatment with 10 μM capsaicin for 1 h. (c and d) Mean concentration‐response curves and mean EC_50_ and *E*_max_ values showing the effect of capsaicin on [Ca^2+^]_o_‐induced relaxations of U46619‐mediated precontracted tone in first‐order vessel segments. (e) Mean concentration‐response curve of capsaicin‐induced relaxation of precontracted tone in first‐order vessel segments. Mean values are ± standard deviation. Each point is from *n* = number of animals with number of vessel segments in brackets. Statistical analysis carried out between control and effect of pretreatment with capsaicin for 1 h on [Ca^2+^]_o_‐induced vasorelaxations. *p* values are in brackets in tables and above data points on graphs.

Figure 2a–d shows that increasing [Ca^2+^]o from 1 to 10 mM induced concentration‐dependent relaxations of precontracted tone to 300 nM U46619 in first‐order arterial segments, with a mean EC_50_ value of about 5–6 mM [Ca^2+^]_o_ and almost complete relaxations (*E*_max_) at 10 mM [Ca^2+^]_o_. These responses are the same as [Ca^2+^]_o_‐induced vasorelaxations previously shown to be inhibited by the calcilytic Calhex‐231 and proposed to be mediated by stimulation of the CaSR (Carlton‐Carew et al., 2024). Figure 2b–d reveal that pretreatment of vessel segments with 10 μM capsaicin for 1 h produced a significant rightward shift in [Ca^2+^]_o_‐induced vasorelaxations of precontracted tone, increasing mean EC_50_ values to about 8 mM and inhibiting *E*_max_ values by about 50%. Figure 2e shows that acute application of capsaicin produced relaxations of precontracted tone with a EC_50_ of about 6 nM and an almost complete relaxation at 100 nM, which suggests that pretreatment with 10 μM capsaicin for 1 h is likely to produce significant stimulation and desensitization of perivascular sensory nerves.

These results provide strong evidence that desensitization of capsaicin‐sensitive perivascular sensory nerves in rat mesenteric arteries greatly reduces [Ca^2+^]_o_‐induced relaxations, indicating a role for the CaSR expressed on perivascular sensory nerves in these responses.

### 3.3 CGRP and sub P mediate [Ca^2+^]_o_‐induced relaxations in rat mesenteric arteries

Release of vasoactive substances such as CGRP, Sub P, and anandamide from perivascular sensory nerves stimulate CGRP, neurokinin 1 (NK1), and cannabinoid (CB_1_) receptors respectively to mediate endothelium‐dependent and ‐independent changes in vascular tone (Aalkjaer et al., 2021; Wescott & Segal, 2013). We therefore investigated if these receptors were involved in mediating [Ca^2+^]_o_‐induced relaxations of precontracted tone.

**FIGURE 3:**
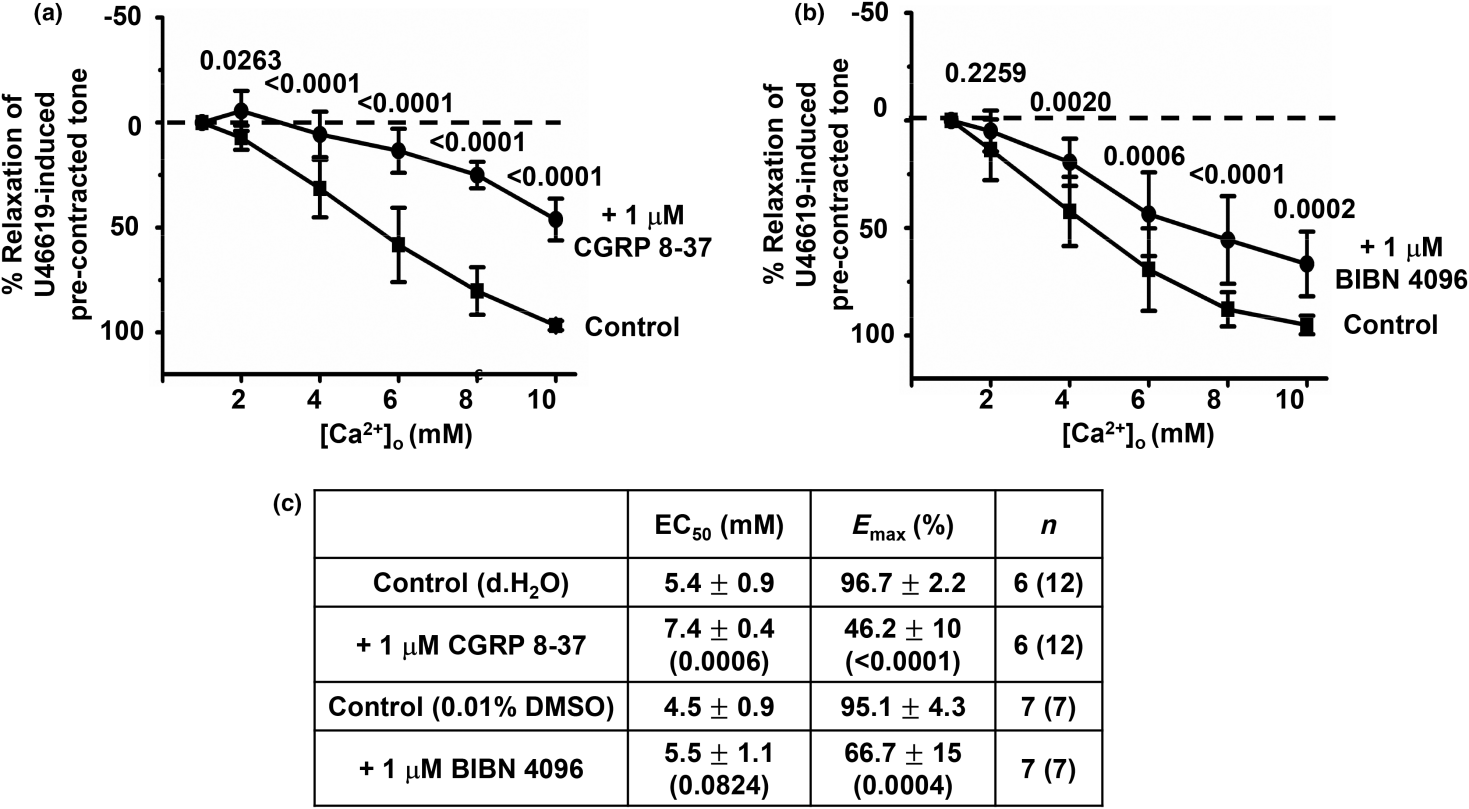
CGRP receptor blockers inhibit [Ca^2+^]_o_‐induced vasorelaxations. (a and b), Mean concentration‐response curves of [Ca^2+^]_o_‐induced relaxations in first‐order vessel segments showing that responses are reduced by 1 μM CGPR 8–37 and 1 μM BIBN 4096. (c), Mean EC_50_ and *E*_max_ values from these experiments. Mean values are ± standard deviation. Each point is from *n* = number of animals with number of vessel segments in brackets. Statistical analysis carried out between effect of [Ca^2+^]_o_ on controls versus in the presence of CGRP 8–37 and BIBN 4096. *p* values are in brackets in tables and above data points on graphs.

**FIGURE 4:**
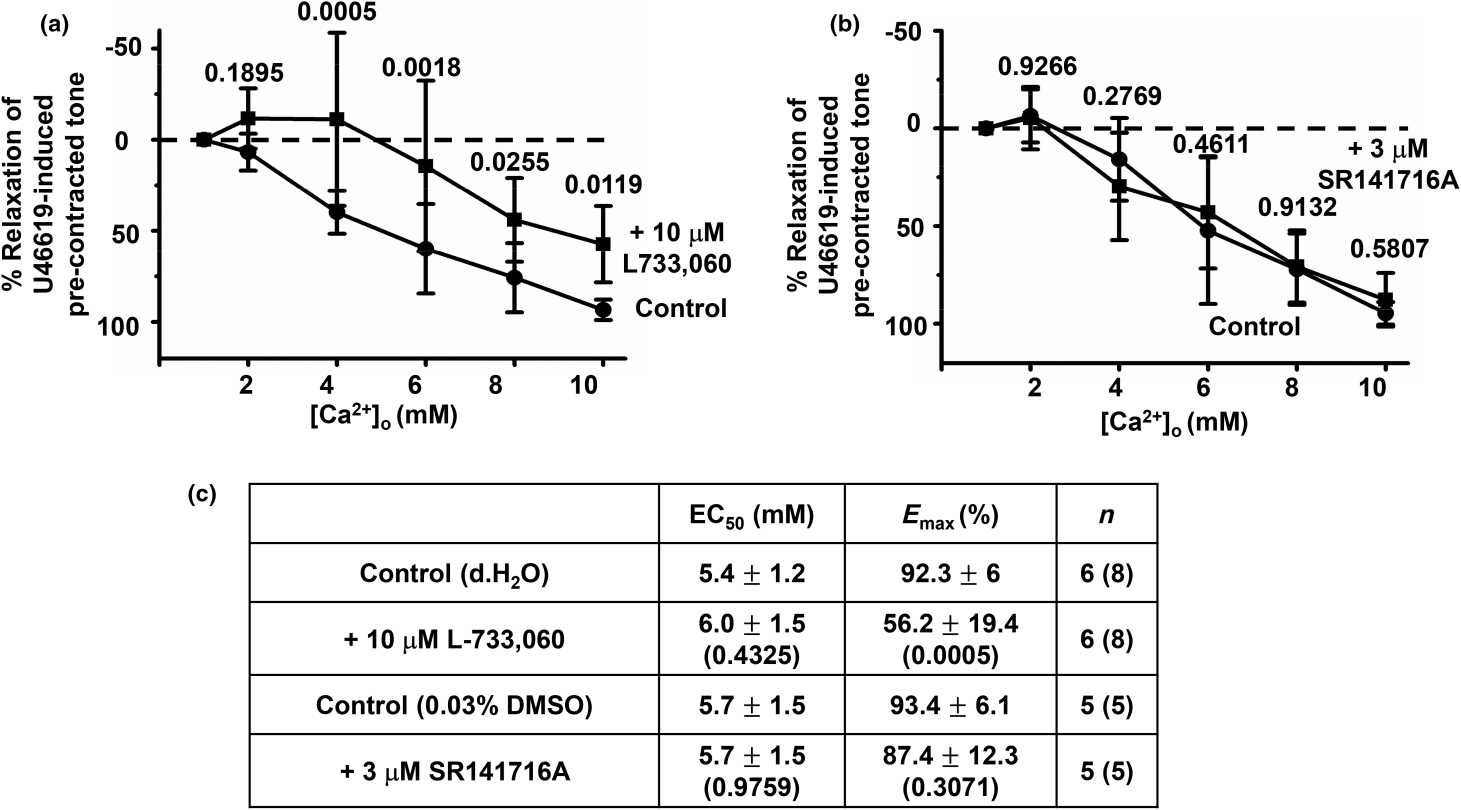
NK1 receptor blocker L733,060 but not the CB_1_ receptor blocker SR141716A reduces [Ca^2+^]_o_‐induced vasorelaxations. (a) Mean concentration‐response curves showing that [Ca^2+^]_o_‐induced relaxations of precontracted tone in first‐order vessel segments are reduced by pretreatment with 10 μM L733,060. (b) Mean data showing that pretreatment with 3 μM SR141716A had no effect on [Ca^2+^]_o_‐induced relaxations in first‐order vessel segments. (c) Mean EC_50_ and *E*_max_ values from these experiments, Mean values are ± standard deviation. Each point is from *n* = number of animals with number of vessel segments in brackets. Statistical analysis carried out between effect of [Ca^2+^]_o_ on controls versus test conditions. *p* values are in brackets in tables and above data points on graphs.

**FIGURE 5:**
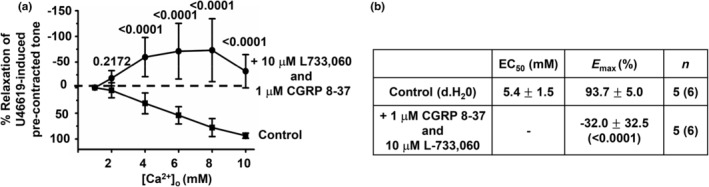
Combination of CGRP and NK1 receptor blockers abolishes [Ca^2+^]_o_‐induced vasorelaxations. (a and b) Mean concentration‐response curves and mean EC_50_ and *E*_max_ values showing that [Ca^2+^]_o_‐induced relaxations in first‐order vessel segments are completely abolished by pretreatment with a combination of both 1 μM CGPR 8–37 and 10 μM L733,060. Instead, increasing [Ca^2+^]_o_ now augmented precontracted tone. Mean values are ± standard deviation. Each point is from *n* = number of animals with number of vessel segments in brackets. Statistical analysis carried out between effect of [Ca^2+^]_o_ on control versus test condition. *p* values are in brackets in tables and above data points on graphs.

Figures 3a–c and 4a,c show that pretreatment with two different CGRP receptor blockers, 1 μM CGRP 8–37 and 1 μM BIBN 4096, and the NK1 receptor blocker 10 μM L733,060 inhibited [Ca^2+^]_o_‐induced relaxations of precontracted tone in first‐order rat mesenteric arteries, respectively. In contrast, Figure 4b,c shows that pretreatment with the CB_1_ receptor blocker 3 μM SR141716A had no effect on [Ca^2+^]_o_‐induced vasorelaxations. Interestingly, Figure 5a,b also shows that pretreatment with 10 μM L733,060 and 1 μM CGRP 8–37 together abolished [Ca^2+^]_o_‐induced relaxations of precontracted tone in arterial segments, with increasing [Ca^2+^]_o_ now producing a marked increase in precontracted tone.

These findings indicate that both CGRP and NK1 receptors, but not CB_1_ receptors, contribute to [Ca^2+^]_o_‐induced relaxations of rat mesenteric arteries.

### 3.4 CGRP and NK1 receptors mediate [Ca^2+^]_o_‐induced vasorelaxations via endothelium‐dependent and ‐independent pathways

We previously suggested that [Ca^2+^]_o_‐induced relaxations of rat mesenteric arteries mediated by the CaSR involved both endothelium‐dependent and independent pathways (Carlton‐Carew et al., 2024), and therefore we investigated the effect of a functional endothelium on involvement of CGRP and NK1 receptors.

**FIGURE 6:**
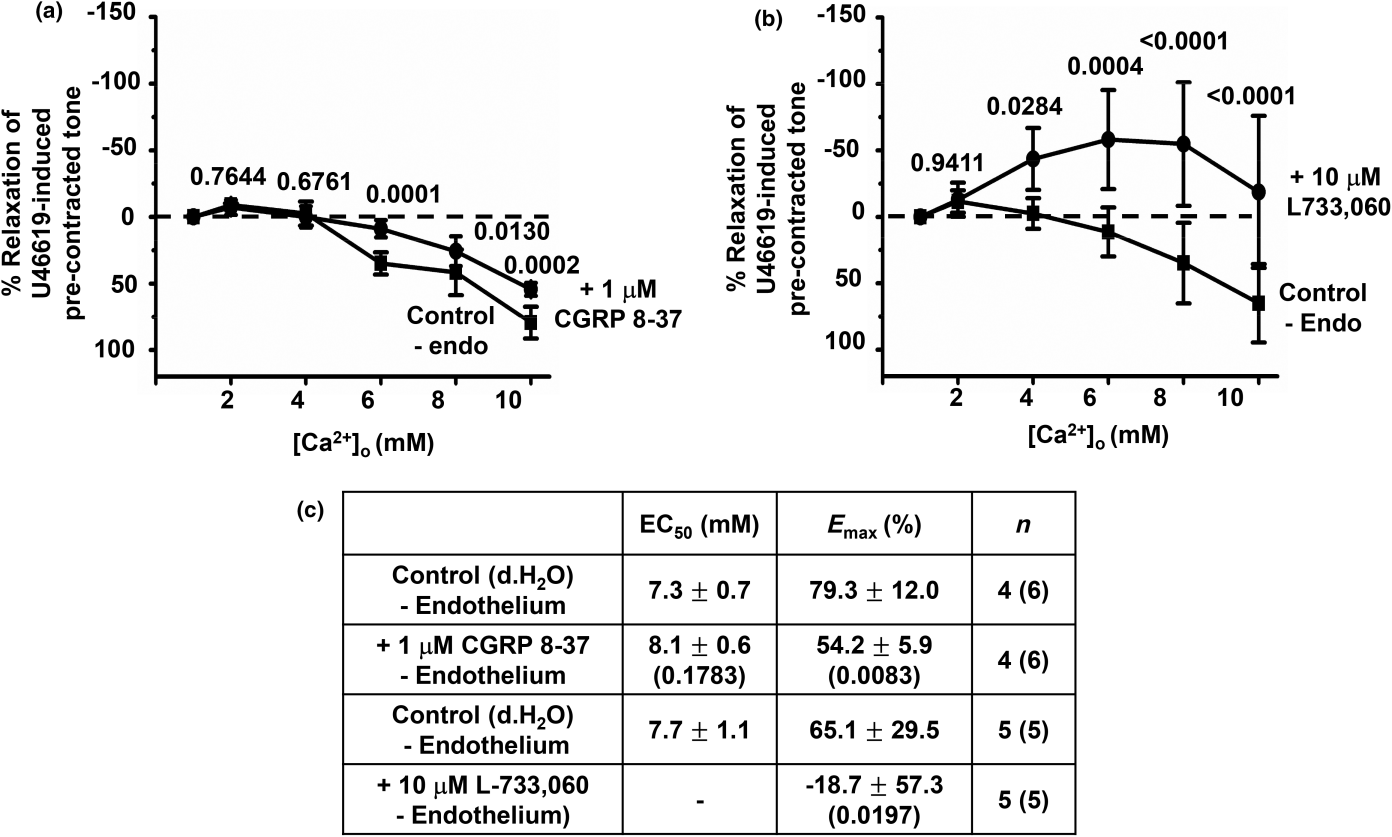
The effect of CGRP and NK1 receptor blockers on [Ca^2+^]_o_‐induced vasorelaxations in the absence of a functional endothelium. (a) Mean concentration‐response curves of [Ca^2+^]_o_‐induced relaxations on precontracted tone showing that pretreatment with 10 μM CGRP 8–37 had a small inhibitory action in first‐order vessel segments without a functional endothelium. (b) Mean concentration‐response curves showing that [Ca^2+^]_o_‐induced relaxations on precontracted tone are completely abolished by pretreatment with 10 μM L733,060 in first‐order vessel segments without a functional endothelium. In these experiments, increasing [Ca^2+^]_o_ augmented precontracted tone. (c) Mean EC_50_ and *E*_max_ values from these experiments. Mean values are ± standard deviation. Each point is from *n* = number of animals with number of vessel segments in brackets. Statistical analysis carried out between effect of [Ca^2+^]_o_ on controls versus test conditions. *p* values are in brackets in tables and above data points on graphs.

Figure 6a–c shows that [Ca^2+^]o‐induced vasorelaxations were partially reduced by removal of a functional endothelium as previously described (see Section 2 and Carlton‐Carew et al., 2024). In addition, Figure 6a,c show that the inhibitory action of 1 μM CGRP 8–37 on [Ca^2+^]_o_‐induced vasorelaxations was greatly reduced in the absence of a functional endothelium (see Figure 3). In contrast, in the absence of a functional endothelium, Figure 6b,c shows that 10 μM L733,060 now completely abolished [Ca^2+^]_o_‐induced vasorelaxations with increasing [Ca^2+^]_o_ inducing a pronounced augmentation of precontracted tone.

These findings suggest that CGRP and NK1 receptors contribute to [Ca^2+^]_o_‐induced vasorelaxations via endothelium‐dependent and ‐independent pathways respectively. In addition, these results further suggest that stimulation of the CaSR by increasing [Ca^2+^]_o_ can mediate both vasorelaxation and vasocontractile actions on precontracted tone in rat mesenteric arteries.

### 3.5 [Ca^2+^]_o_‐induced vasorelaxations mediated by perivascular sensory nerves involve endothelium‐dependent NO production

We have previously proposed that [Ca^2+^]_o_‐induced relaxations in rat mesenteric arteries are mediated by a endothelium‐dependent pathway involving NO production and an endothelium‐independent pathway involving stimulation of K_ATP_ channels in VSMCs (Carlton‐Carew et al., 2024). Therefore in a final series of experiments studied that role of eNOS and K_ATP_ channels in [Ca^2+^]_o_‐induced vasorelaxations following pretreatment with capsaicin.

**FIGURE 7:**
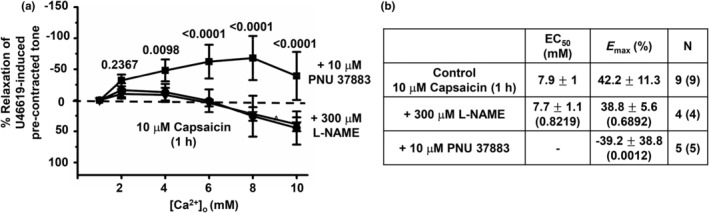
In the absence of a functional endothelium, neurokinin receptor and K_ATP_ channel blockers inhibit [Ca^2+^]_o_‐induced vasorelaxations. (a and b) Mean concentration‐response curves and mean EC_50_ and *E*_max_ values showing that pretreatment with 10 μM PNU 37883 abolished [Ca^2+^]_o_‐induced relaxations in first‐order vessel segments treated with 10 μM capsaicin for 1 h whereas pretreatment with 300 μM L‐NAME had no effect. Mean values are ± standard deviation. Each point is from *n* = number of animals with number of vessel segments in brackets. Statistical analysis carried out between effect of [Ca^2+^]_o_ on controls versus test conditions. *p* values are in brackets in tables and above data points on graphs.

Figure 7a,b shows that following pretreatment with 10 μM capsaicin for 1 h, the remaining [Ca^2+^]_o_‐induced relaxations of precontracted tone were not affected by the eNOS inhibitor 300 μM L‐NAME but were completely abolished by 10 μM PNU 37883. Moreover, in the presence of 10 μM PNU 37883 increasing [Ca^2+^]_o_ augmented precontracted tone.

## 4 DISCUSSION

The present work shows that increasing [Ca^2+^]_o_ induces relaxation of precontracted tone in rat mesenteric arteries, which is likely mediated by stimulation of the CaSR on perivascular sensory nerves and subsequent activation of CGRP and NK1 receptor‐mediated pathways involving endothelium‐dependent NO production and endothelium‐independent activation of K_ATP_ channels, respectively.

### 4.1 CaSRs are expressed in perivascular nerves in rat mesenteric arteries

We demonstrate using immunofluorescence studies that the synaptic terminal marker synaptophysin and the CaSR colocalise in mesh‐like networks of perivascular nerves, likely containing sympathetic and perivascular sensory nerves, in the adventitial layer of first‐order rat mesenteric artery segments. These results support our recent study showing co‐localisation between synaptophysin and the CaSR in the adventitial layer of transverse sections of rat mesenteric arteries (Carlton‐Carew et al., 2024), and previous work showing CaSR expression in perivascular sensory nerves of rat mesenteric arteries through the presence of CaSR mRNA in dorsal root ganglia (Bukoski et al., 1997; Wang & Bukoski, 1998) and CaSR staining and co‐localisation with the nerve cell marker neural cell adhesion molecule (NCAM) (Bukoski et al., 2002; Mupanomunda et al., 1998; Wang & Bukoski, 1998).

### 4.2 Increasing [Ca^2+^]_o_ produces relaxations in rat mesenteric arteries

Increasing [Ca^2+^]_o_ from 1 to 10 mM induced concentration‐dependent relaxations of precontracted tone by a thromboxane agonist in first‐order rat mesenteric arteries, which had a mean EC_50_ value of about 5–6 mM [Ca^2+^]_o_ and almost full relaxation at 10 mM [Ca^2+^]_o_. These findings are the same as [Ca^2+^]_o_‐induced relaxations in rat mesenteric arteries previously described which were inhibited by the calcilytic Calhex‐231 and thus likely to involve stimulation of the CaSR (Carlton‐Carew et al., 2024). This is further supported by earlier studies showing CaSR‐mediated relaxations by increasing [Ca^2+^]_o_ in rat, rabbit, and mouse mesenteric arteries (Awumey et al., 2008, 2013; Bukoski et al., 1997, 2002; Dora et al., 2008; Greenberg et al., 2017, 2019; Greenberg, Jahan, et al., 2016; Greenberg, Shi, et al., 2016; Ishioka & Bukoski, 1999; Mupanomunda et al., 1998, 1999; Thakore & Ho, 2011; Wang & Bukoski, 1998, 1999; Weston et al., 2005, 2008). It is important to acknowledge that other non‐CaSR‐mediated mechanisms may also contribute to the observed [Ca^2+^]_o_‐induced vasorelaxations, for example [Ca^2+^]_o_ is proposed to modulate K_ir_ channel and Na^+^/K^+^‐ATPase activities which are known to regulate vasorelaxation (Hangaard et al., 2015).

Previously, CaSR‐mediated relaxations of first‐ and second‐order rat mesenteric arteries precontracted with α_1_‐adrenoceptor stimulants noradrenaline or phenylephrine had a mean EC_50_ value of about 2–3 mM, with 5–6 mM [Ca^2+^]_o_ producing near full relaxation (Bukoski et al., 1997; Ishioka & Bukoski, 1999; Mupanomunda et al., 1998, 1999; Wang & Bukoski, 1998, 1999), which are lower than the values recorded in our current and previous studies (Carlton‐Carew et al., 2024). It is unclear why [Ca^2+^]_o_‐induced vasorelaxations recorded in our studies are less sensitive to [Ca^2+^]_o_, but it might be due to this study using the thromboxane receptor agonist U46619 to produce precontracted tone and not an α_1_‐adrenoceptor stimulant.

Plasma [Ca^2+^]_o_ is closely regulated between 1 and 2 mM (Brown & Macleod, 2001) and a pertinent question is whether [Ca^2+^]_o_‐induced vasorelaxations with an EC_50_ of 5–6 mM are physiological? The CaSR in the vasculature is likely activated by paracrine Ca^2+^ signaling within interstitial spaces produced by active Ca^2+^ extrusion systems in VSMCs and VECs such as the plasma membrane Ca^2+^‐ATPase and Na^+^/Ca^2+^ exchanger (Dora et al., 2008), which probably produce localized [Ca^2+^]_o_ rises substantially higher than plasma levels. In a similar process, external K^+^ concentration ([K^+^]_o_) rises to over 10 mM within interstitial spaces in rat hepatic and mesenteric arteries due to opening of K^+^ channels and changes in Na^+^/K^+^‐ATPase activity, with this rise of [K^+^]_o_ inducing vasorelaxations by acting as an endothelium‐derived relaxant factor (Edwards et al., 1998; Weston et al., 2008). It would be useful to confirm these ideas by measuring [Ca^2+^]_o_ changes in the interstitial spaces of the vascular using Ca^2+^‐sensitive microelectrodes (Messerli & Smith, 2010).

### 4.3 [Ca^2+^]_o_‐induced vasorelaxations are partially mediated by capsaicin‐sensitive perivascular sensory nerves

In our previous study, we showed that CaSR‐induced relaxations in rat mesenteric arteries partially require a functional endothelium, which means that the CaSR involved in these responses is likely expressed upstream on perivascular nerves and/or directly on VECs (Carlton‐Carew et al., 2024). To investigate the role of CaSR on perivascular sensory nerves, the present study investigated the effect of pretreating arteries with capsaicin on [Ca^2+^]_o_‐induced relaxations of precontracted tone, as capsaicin activates and desensitizes TRPV1‐sensitive perivascular sensory nerves to prevent release of vasoactive substances like CGRP, Sub P, and anandamide (Aalkjaer et al., 2021; Wescott & Segal, 2013). Pretreatment with capsaicin for 1 h produced a significant, but not complete, reduction of vasorelaxations induced by 1–10 mM [Ca^2+^]_o_ with associated increased and decreased mean EC_50_ and *E*_Max_ values, respectively. These findings provide strong evidence that stimulation of the CaSR on perivascular sensory nerves contribute to [Ca^2+^]_o_‐induced relaxations of precontracted tone in rat mesenteric arteries. The partial inhibitory action of capsaicin on [Ca^2+^]_o_‐induced vasorelaxations may reflect incomplete desensitization of TRPV1‐sensitive perivascular sensory nerves and/or expression of the CaSR on other cell types such as capsaicin‐insensitive perivascular nerves and VECs.

### 4.4 Role of CGRP and NK1 receptors in [Ca^2+^]_o_‐induced vasorelaxations

Reduction of [Ca^2+^]_o_‐induced vasorelaxations by pretreatment with capsaicin indicates that CaSR‐mediated release of vasoactive substances from TRPV1‐sensitive perivascular sensory nerves might be involved. Our results show that the CGRP receptor blockers CGRP 8–37 and BIBN 4096 and the NK1 receptor blocker L733,060 partially reduced [Ca^2+^]_o_‐induced relaxations of precontracted tone when applied alone to arterial segments whereas the CB_1_ receptor blocker SR141716A had no effect. CGRP 8–37 was slightly more effective at inhibiting [Ca^2+^]_o_‐induced relaxations than BIBN 4096, which may due to drug efficacy acting at the CGRP receptor (known to be composed of CLR and RAMP1) (Liang et al., 2018). Importantly, pretreatment with CGRP 8–37 and L733,060 together completely abolished [Ca^2+^]_o_‐induced vasorelaxations. These results strongly indicate that CaSR‐mediated CGRP and NK1 receptor‐mediated pathways are responsible for [Ca^2+^]_o_‐induced relaxations in rat mesenteric arteries.

### 4.5 CGRP and NK1 receptors mediate [Ca^2+^]_o_‐induced vasorelaxations via endothelium‐dependent and ‐independent pathways involving eNOS and K_ATP_ channels respectively

The inhibitory effect of CGRP 8–37 on [Ca^2+^]_o_‐induced vasorelaxations was markedly reduced in arterial segments containing a nonfunctional endothelium, which suggests that CGRP receptor‐mediated contributions to [Ca^2+^]_o_‐induced vasorelaxations predominantly involve an endothelium‐dependent pathway. Moreover, [Ca^2+^]_o_‐induced vasorelaxations in the presence of capsaicin were unaffected by the eNOS inhibitor L‐NAME, which was previously shown to significantly reduce [Ca^2+^]_o_‐induced vasorelaxations confirming an important role for NO production in these responses (Carlton‐Carew et al., 2024). Together, these findings suggest that stimulation of CaSR on perivascular sensory nerves might induce the release of CGRP and stimulation of CGRP receptor‐mediated NO production in VECs leading to relaxation of VSMCs. These proposals support our previous ideas that CaSR‐mediated eNOS activity, NO production, and subsequent stimulation BK_Ca_ channels in VSMCs contribute to [Ca^2+^]_o_‐induced relaxations in rat mesenteric arteries (Carlton‐Carew et al., 2024).

In contrast, the inhibitory effect of L733,060 on [Ca^2+^]_o_‐induced relaxations on precontracted tone remained in arterial segments containing a nonfunctional endothelium. Moreover, in the presence of capsaicin, the K_ATP_ channel blocker PNU 37883 completely abolished the remaining [Ca^2+^]_o_‐induced vasorelaxations. These findings may suggest that CaSR‐mediated release of Sub P and stimulation of NK1‐mediated K_ATP_ channel activation in VSMCs causes vasorelaxations, further supporting our previous findings that an endothelium‐independent role for K_ATP_ channels is critical in mediating CaSR‐induced relaxations in rat mesenteric arteries (Carlton‐Carew et al., 2024). These results are interesting as Sub P is generally thought to act at NK1 receptors on VECs to produce vasodilatation and at NK1 receptors on VSMCs to produce vasoconstriction (Wescott & Segal, 2013).

In the presence of capsaicin PNU 37883 completely abolished [Ca^2+^]_o_‐induced vasorelaxations. These findings raise the idea that desensitization of TRPV1 channels on perivascular sensory nerves is effective at reducing CGRP release and CGRP receptor‐mediated NO production but less effective at reducing Sub P release and NK1 receptor‐mediated K_ATP_ channel pathway. It is unclear why this might occur. It is possible that the CaSR is located on different populations of perivascular sensory nerves that release either CGRP or Sub P, or that CGRP and Sub P are released from the same nerves but that Sub P release is coupled to TRPV1‐independent pathways such as involvement of TRPA1 channels which are also found in perivascular nerve terminals (Aalkjaer et al., 2021; Wescott & Segal, 2013). It may also be that stimulation of both TRPV1 and TRPA1, often co‐localized with each other in perivascular sensory nerves, and perhaps other molecules are required to release Sub P. In future studies it would be useful to investigate the relationship between perivascular sensory nerves and expression of the CaSR, CGRP, and Sub P content, and TRPV1 and TRPA1 channels following capsaicin pretreatment.

In the presence of CGRP 8–37 and L733,060 added together, increasing [Ca^2+^]_o_ augmented precontracted tone. In addition, [Ca^2+^]_o_‐induced increases in precontracted tone were observed when L733,060 was applied in the absence of a functional endothelium, presumably because removal of a functional endothelium also prevented CGRP receptor‐mediated actions. Furthermore, [Ca^2+^]_o_‐induced increases in precontracted tone were recorded when PNU 37883 were applied separately following pretreatment with capsaicin, again likely because capsaicin had prevented CGRP‐receptor mediated actions. In all these conditions, these vasocontractile effects are likely due to stimulation of the CaSR on VSMCs and activation of the Gq‐coupled receptor contractile pathway as previously suggested (Greenberg, Shi, et al., 2016; Li et al., 2011; Schepelmann et al., 2016; Wonneberger et al., 2000). It would be interesting to investigate these Ca^2+^‐mediated vasoconstrictions using pharmacological, for example, Calhex‐231, and molecular approaches. These findings further indicate that CaSR‐mediated effects on vascular tone are likely to involve both relaxant and contractile actions in the same vascular bed. Moreover, the ability of pharmacological agents that modulate both CGRP‐ and Sub P‐mediated pathways to complete abolish Ca^2+^‐induced vasorelaxations further suggests that Ca^2+^ acting at CaSR on perivascular nerves and not via non‐CaSR actions is likely to produce these findings.

### 4.6 Comparisons with previous studies on [Ca^2+^]_o_‐induced vasorelaxations in rat mesenteric arteries

This present study supports earlier findings on the effects of stimulating the CaSR in rat mesenteric arteries which showed that [Ca^2+^]_o_‐induced vasorelaxations were abolished by phenolic destruction of perivascular nerves and partially reduced by chronic pretreatment with capsaicin which also reduced CGRP‐ and CaSR‐sensitive nerve staining (Bukoski et al., 1997; Mupanomunda et al., 1998). Moreover, these studies and our recent findings also showed that [Ca^2+^]_o_‐induced vasorelaxations are partially mediated by an endothelium‐dependent pathway, and that hyperpolarisations of VSMCs through activation of K^+^ channels, including BK_Ca_ channels, have a pivotal role (Bukoski et al., 1997; Carlton‐Carew et al., 2024; Ishioka & Bukoski, 1999).

However, there are significant differences between previous findings and our work. In previous studies, inhibitors of eNOS, CGRP, and NK1 receptors (Bukoski et al., 1997), and K_ATP_ channels (Ishioka & Bukoski, 1999) all had no effect on [Ca^2+^]_o_‐induced vasorelaxations and a CB_1_ receptor blocker reduced [Ca^2+^]_o_‐induced vasorelaxations (Ishioka & Bukoski, 1999). These results are opposite to our present and recent findings (Carlton‐Carew et al., 2024). Instead, these previous works proposed that [Ca^2+^]_o_‐induced relaxations in rat mesenteric arteries were mediated by the CaSR on perivascular nerves leading to production and release of cytochrome P_450_‐ and phospholipase A_2_‐mediated metabolites and anandamide which directly and indirectly stimulate BK_Ca_ channels in VSMCs (Awumey et al., 2008) and also the CaSR expressed in VECs which lead stimulation of IK_Ca_ channels and hyperpolarisations (Weston et al., 2005). It is unclear why there might be such differences between findings on the cellular mechanisms that underlie stimulation of CaSRs on perivascular nerves and relaxations in the same species and vascular bed. These issues affirm the importance of investigating the actions of the CaSR in the human vasculature.

## 5 CONCLUSION

**FIGURE 8:**
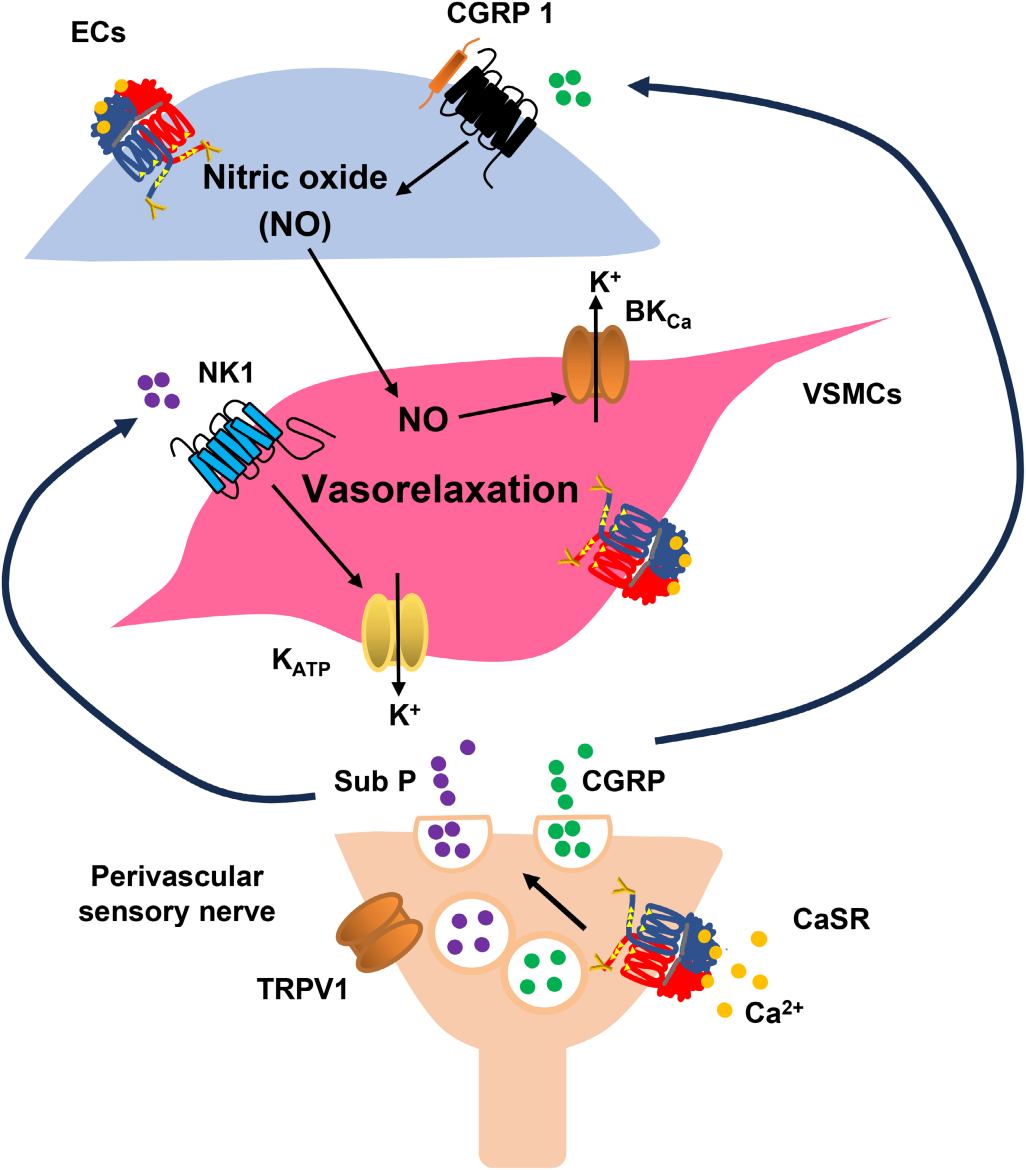
Schematic diagram of proposed mechanisms underlying CaSR‐induced vasorelaxations in rat mesenteric arteries. It is proposed that stimulation of the CaSR on TRPV1‐containing perivascular sensory nerve terminals by an increase in [Ca^2+^]_o_ leads to release of calcitonin‐gene‐related peptide (CGRP) and substance P (Sub P). CGRP acts at CGRP1 receptors on endothelial cells (ECs) to induce nitric oxide (NO) generation and NO‐mediated stimulation of BK_Ca_ channels in vascular smooth muscle cells (VSMCs). In contrast, Sub P acts at NK1 receptors on VSMCs to stimulate K_ATP_ channels. It is likely that activation of BK_Ca_ and K_ATP_ channels leads to vasorelaxations through K^+^ efflux and, hyperpolarisations. As described in the discussion, the CaSR is also expressed in ECs and VSMCs and that these CaSR‐mediated mechanisms may contribute to vasorelaxations.

The present study clearly indicates that stimulation of the CaSR on perivascular sensory nerves by increasing [Ca^2+^]_o_ induces relaxation of rat mesenteric arteries (Figure 8). Our findings suggest for the first time that [Ca^2+^]_o_‐induced vasorelaxations occur via CGRP and NK1 receptor‐mediated pathways: stimulation of the CaSR on capsaicin‐sensitive perivascular sensory nerves leads to CGRP release and activation of CGRP receptors to induce eNOS activity and NO production in VECs, and stimulation of the CaSR, possibly on capsaicin‐insensitive perivascular nerves, leads to Sub P release and activation of NK1 receptors and K_ATP_ channels on VSMCs. The activation of BK_Ca_ and K_ATP_ channels is likely to lead to vasorelaxations through K^+^ efflux and hyperpolarisations.

## AUTHOR CONTRIBUTIONS

S.R.E.C‐C performed and analyzed experiments. S.R.E.C‐C, H.Z.E.G, I.A.G, and A.P.A conceived the experimental design. A.P.A. wrote the manuscript. All authors contributed to the preparation of the manuscript, and critically advised and agreed to the final submitted article.

## FUNDING INFORMATION

This work was supported by a British Heart Foundation PhD Studentships for H. Z. E. Greenberg (FS/13/10/30021 to A.P.A) and S.R.E.C‐C (FS/17/40/32942 to A.P.A); and by the Biotechnology and Biological Sciences Research Council (BB/J007226/1 to A.P.A).

## CONFLICT OF INTEREST STATEMENT

None declared.

## Data Availability

The data that support the findings of this study are available from the corresponding author upon reasonable request.
